# Virtual Reality as a Tool to Study the Influence of the Eating Environment on Eating Behavior: A Feasibility Study

**DOI:** 10.3390/foods10010089

**Published:** 2021-01-05

**Authors:** James H. Oliver, James H. Hollis

**Affiliations:** 1 Virtual Reality Applications Center, Iowa State University, Ames, IA 50011, USA; oliver@iastate.edu; 2 Department of Food Science and Human Nutrition, Iowa State University, Ames, IA 50011, USA

**Keywords:** virtual reality, mastication, food intake, perception

## Abstract

In this manuscript, we describe a new approach to study the effect of the eating environment on food intake and eating behavior using virtual reality technology. Fifteen adults consumed pizza rolls in two virtual reality (VR) environments: a restaurant and a table in an empty room. The participants’ food intake, eating parameters (e.g., masticatory parameters and eating rate), and their sensory evaluation of the test food was measured. The participants’ sense of presence (the feeling of being in the virtual environment) and markers of arousal were also measured. There was no statistical significant difference in food intake or the sensory evaluation of the test food. In the restaurant condition, participants used fewer masticatory cycles before swallowing but there was no effect on eating rate or maximum bite force. Participants experienced a greater sense of presence when they were in the pizza restaurant scene. Moreover, their heart rate and skin temperature were higher in the restaurant condition. This study suggests that VR could be developed as a new tool to study the effect of the eating environment on food intake and eating behavior.

## 1. Introduction

Data from the National Health and Nutrition Examination Survey indicate that eating away from home (EAFH) accounts for 42% of all food expenditures and 32% of all calories consumed in the United States, and approximately 50% of adults consume at least three meals away from home each week [1]. The regularity of EAFH has public health implications as energy intake is higher than when eating at home, and the frequency of EAFH may be a contributory factor to the obesity epidemic [2,3,4]. A better understanding of the factors that influence food intake in different eating environments would likely aid the development of new strategies to reduce the number of individuals with obesity.

Several food-intrinsic factors, such as portion size, energy density, and the taste/palatability, have been implicated as contributors to higher energy intake when EAFH [5]. However, accumulating data indicates that food-extrinsic factors (factors that are related to the food but are not part of the food itself) also influence meal size. A growing body of evidence suggests that food-extrinsic factors can influence the sensory perception of a food [6]. For instance, studies have demonstrated that the background noise or music can make a wine taste fruitier and smoother [7]. Different environmental sounds have also been shown to alter the perception of bitter, roasted, and cocoa notes of chocolate gelati [8]. Other studies found that changing the color of a plate, bowl, or cup changes the perceived sweetness of popcorn [9], yogurt [10], or coffee [11]. Wang et al. [12] found that environmental odors influence the perceived sweetness of foods. In addition, Hasenbeck et al. [13] reported that the color and intensity of lighting modulates the willingness to eat a food or the meal size. These observations are of interest as food palatability is an influence on meal size [14]. In addition, a better understanding of how food-extrinsic factors influence the perception of food may provide opportunities to reformulate foods to make them less energy-dense and reduce energy intake at a meal. For instance, if the color of a plate increases the sweet taste of a food, the amount of a caloric sweetener in a meal can be reduced without reducing palatability. 

Any environment has emotion-eliciting qualities [15]. Moreover, how an individual responds in that environment is influenced by the emotions evoked by that environment [16,17]. For instance, the shopping environment in retail stores influences browsing, purchase intentions, and shopping time [18,19]. Emotional states are accompanied by states of physiological arousal, which can be measured using methods such as heart-rate monitors, skin conductance, or temperature [20]. Studies indicate that ambient factors in the eating environment can increase arousal [21,22]. Moreover, a higher heart rate during a meal was a significant predictor of the amount of food consumed [21,23]. Based on this evidence, it is possible that different restaurant environments may influence food intake through an effect on the strength of the emotional response. A better understanding of how eating environments influence emotions may provide new insights into why some individuals overeat at restaurants [24].

Laboratory studies show that the microstructure of eating (e.g., eating rate, number of chewing cycles, bite size, etc.) influences meal size and postprandial appetite. For instance, a number of studies have reported that differences in eating rate [25] or masticatory parameters influence meal size or postprandial satiety [26,27,28]. Studies of free-living individuals report that a faster eating rate is associated with increased body mass index and risk of weight gain [24,29]. While many studies have shown that food-intrinsic factors influence the microstructure of eating [30,31] little is currently known about how the eating environment influences eating parameters. Research to determine if the eating environment influence eating rate or the microstructure of eating may lead to new insights into why some individuals overeat at restaurants.

A significant barrier to understanding how eating in restaurants influences meal size (or putative causative influences on meal size) is gaining access to restaurants to conduct research studies. Even if access to a restaurant can be obtained, the ability to manipulate the restaurant environment to determine its effect on eating or the sensory perception of foods or eating may be limited. It is likely that different types of restaurant environments may elicit divergent responses, meaning that results from one type of restaurant cannot be generalized across all restaurants. A possible alternative approach is to use computer-generated 3D models of restaurants to create an immersive and interactive virtual reality (VR) environment. As these environments are computer-generated, all aspects of the virtual restaurant environment can be manipulated through software and any type of restaurant can be modeled. 

Currently, two main forms of experiencing VR content are in use: the head-mounted display (HMD) (e.g., Oculus Rift or HTC Vive) or the CAVE (Cave Automatic Virtual Environment) systems (e.g., Mechdyne CAVE). CAVE systems typically use projectors to display stereoscopic computer-generated images on several walls (and/or floor and ceiling surfaces) to create a sense of immersion in a virtual world. In contrast to an HMD, which provides distinct images to left and right eyes while fully occluding natural ambient light, a CAVE system uses see-through shutter glasses to differentiate frame-sequential images on the walls to produce the stereoscopic effect. Thus, CAVE systems offer several advantages over HMDs as a method to study eating behavior, including perception of self (i.e., the user sees and perceives their own body), which enhances the “naturalness” of eating. A CAVE also eliminates the need to spatially register the physical food with its virtual counterpart, which is necessary with an HMD implementation. However, a drawback to CAVE systems is that that they are relatively expensive, require dedicated space and resources, and may be complex to implement. While HMDs impinge on natural eating behavior, they are inexpensive and require markedly fewer resources to operate. Consequently, if individuals can eat while wearing an HMD, this may offer a relatively inexpensive method to study eating behavior in different simulated eating environments. A recent study found that sensory testing could successfully be conducted in VR using an HMD in which participants were required to eat [32], suggesting the utility of this approach for studying aspects of eating behavior. Moreover, VR has also been used to better understand food selection at a buffet [33] and the amount of food served at a buffet [34]. A virtual cafeteria has also been reported to be a useful method to educate individuals on portion size [35].

When designing effective VR scenes to study eating behavior, it is essential to create a sense of presence so that users suspend disbelief and believe they are actually present in the VR environment, and respond to stimuli as they would in equivalent real-life situations [36,37]. Despite its importance, there is no generally accepted measure of “presence”, although questionnaires are currently the preferred method [38]. To date, several physiological markers have been proposed as markers of presence, including heart rate, heart rate variability, skin conductance, skin temperature, and EEG, although their utility has not been clearly established [38,39]. To maximize the usefulness of immersive VR to study the effects of eating environment on eating behavior, it is vital that a participant feels that they are actually present in the simulated eating environment. 

The aim of this manuscript is to describe an approach that may be used to study the effect of the eating environment on food intake and eating behavior using a VR-HMD. We provide exploratory data regarding the influence of two different eating environments on subjective presence, food intake, masticatory parameters, heart rate, skin temperature, and the sensory evaluation of the test food.

## 2. Materials and Methods 

**Figure 1:**
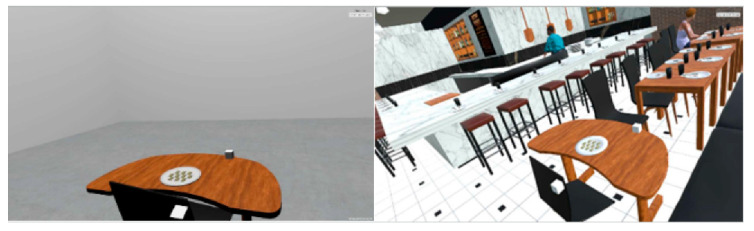
(**left**) The minimal blank VR scene and (**right**) the restaurant VR scene.

Potential participants were contacted via email or word of mouth. Participants were recruited from Iowa State University staff and students, and from the local community. All test sessions were conducted at the Virtual Reality Applications Center at Iowa State University. Fifteen adults were recruited subject to the following inclusion criteria: aged 18–50 years, body mass index (BMI) between 19.9 and 29.9, reported the test foods to be palatable (>5 on a 9 point scale). Potential participants were excluded if they had a history of seizures, a history of motion sickness, acute disease, used tobacco products (due to potential suppressive effect on appetite), had had dental treatment in the previous three months (due to a potential effect on masticatory parameters), were allergic to any of the ingredients in the test foods, or consumed more than 21 alcoholic drinks per week (due to potential stimulatory effect on appetite). Previous experience with VR was not a criteria for inclusion or exclusion and we did not quantify previous use of VR headsets. Participants who met the eligibility criteria (nine male, six female; mean age = 30 years (SD (Standard Deviation) = 4) and mean BMI = 23 kg m^−2^ (SD = 1)) were briefed about the study and asked to sign an informed consent document. The participants were then randomized to a treatment order. This study used two treatments: a restaurant scene and a blank scene that contained only a table with a plate (Figure 1).

### 2.1. Procedure

The study was conducted in accordance with the Declaration of Helsinki, and the protocol was approved by the Institutional Review Board of Iowa State University (#17-245).

Participants were asked to report to the laboratory on two occasions, separated by at least one week, at their usual lunchtime. On reporting to the laboratory, an Empatica E4 wristband (Empatica, Boston, MA, USA) was placed on the participant’s nondominant wrist. Four surface electrodes were attached to the left and right masseter muscles, which were located by palpitation in a bipolar configuration. The surface electrodes were connected to a Biopac MP36R (BIOPAC Systems Inc., California, CA, USA) that gathered data regarding masseter muscle activation. The participant was then asked to sit quietly for 5 min so that baseline heart rate and skin temperature could be measured. A virtual reality head-mounted display (VR-HMD) was then placed on their head and displayed the relevant VR scene. 

To facilitate eating while wearing a HMD, this present study used an infrared camera (Leap Motion, which models the movement of the user’s real hands onto the VR space so that they can locate and interact with objects in the virtual environment. Objects (table, plate, and pizza bites) in virtual space were mapped onto the location of the same objects in the real world using Vive trackers attached to the chair and table. Thus, when the user touched the table, chair, plate, or food in the virtual environment, they touched a real-life table, chair, plate, or pizza bite. During debriefing, participants did not report difficulty locating the food, although placing the food in the mouth took a minor amount of practice and concentration.

**Figure 2:**
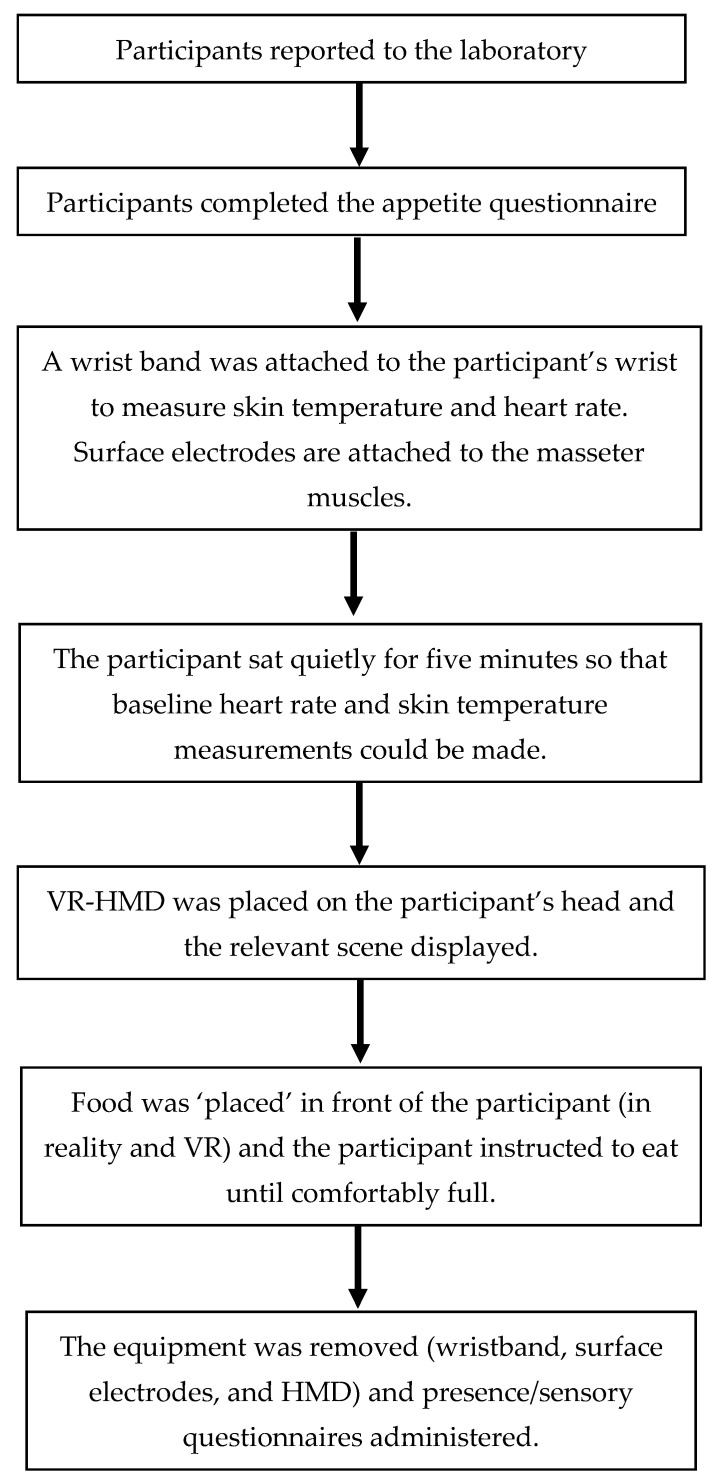
A simple schematic of the study procedure. Specific details of the procedures are provided in Section 2.1.

The test foods were represented in VR using a 3D model of the test food arranged in rows on a plate. The participant was initially asked to reach out with both hands to touch the plate in a circumferential manner. This had the effect of acclimating the participant to the virtual representation of their hands while they also (inadvertently) helped to spatially register the physical plate with its virtual counterpart. The participant did not see the food on the plate before the HMD was placed on their head and could not locate the pizza bites through memory. When the participant touched the test food in the virtual world they touched the real test food, allowing them to locate and pick up the test food. The participant was instructed to eat the test food until they felt comfortably full, and to access the test food (pizza rolls) in a specific order, e.g., “Start with the upper left-most roll.” Each time a pizza roll was accessed, while the participant was consuming it, the researcher would press a key on the keyboard to remove one pizza roll from the virtual plate. This provided the participant with a visual cue of how much they had eaten. The participant was instructed to eat the test food until they felt comfortably full. When the participant reported that they had finished eating, the VR-HMD, Empatica wristband, and the surface electrodes were removed. The participant completed a questionnaire regarding their feelings of presence and experiences in the VR environment and their ratings of the test food attributes. The participant was then free to leave the laboratory. A diagram of the flow through the study is provided in Figure 2.

### 2.2. Virtual Reality

A HTC Vive (HTC Corporation, Taoyuan City, Taiwan) HMD was used for this study. This headset provides 110 degrees of viewing at a resolution of 1080 × 1200 pixels per eye. The headset has a refresh rate of 90 Hz. 

### 2.3. Virtual Reality Scenes

The virtual reality scenes were created using the Unity Game Engine (version 2017.3.0f3). The blank scene consisted of a table in a white scene, while the restaurant scene was a computer-generated representation of a modern restaurant (Figure 1). The restaurant scene contained many elements of a modern restaurant, including televisions displaying videos of sports games, wait staff moving around the scene, other restaurant customers present in the scene, and a street scene with moving pedestrians and cars. It also included normal ambient restaurant sounds including kitchen sounds and muffled nearby conversations. HTC Vive Trackers (HTC Corporation, Taoyuan City, Taiwan) were attached to a chair and table so that when these were moved in the real world, they also moved in the virtual world. 

### 2.4. Test Food

Participants were served a portion of ten Totinos Cheese Pizza Rolls (Totino’s Cheese Frozen Rolls, General Mills, MN, USA). The pizza rolls were cooked using the manufacturer’s instructions and allowed to cool to 60 °C before serving. Each pizza roll provided 35 kcal. The participant could request another serving of pizza rolls if they completed the first serving. The second serving was presented in the same way as the first serving. The pizza roll was represented in the virtual world by a shape that had similar dimensions and color but was not a photorealistic representation of an actual pizza roll. 

### 2.5. Questionnaires

This study used a questionnaire that was developed over a number of studies by Slater and colleagues and used in Reference [40]. Participants answered the six questions relating to presence in the virtual environment and responses were captured using a 7 point Likert scale. The participant responses to each question were combined to form an average of the presence question responses. Subjective appetite was determined using a questionnaire used in previous studies to measure appetite [41]. The participants answered the following questions: “How hungry are you right now?” “How full do you feel right now?” “How strong is your desire to eat right now?” and “How much could you eat right now?” Participant responses were captured on a 100 mm visual analogue scale (VAS). The VAS was anchored by “not at all” or “nothing at all” on the left side and “as hungry/full/strong has it as ever been” or a “large amount” on the right side. After the meal, the participants were asked to answer the questions, (a) “How palatable was the meal?” (b) “How salty was the meal?” (c) “Did you enjoy the texture of the meal?” and (d) “Did you find the meal acceptable?” on a 9-point Likert scale. The scale was anchored with “Dislike extremely” and “Like extremely”, “Not at all salty” and “Extremely salty”, “Very unpleasant” and “Very pleasant”, and “Extremely unacceptable” and “Extremely acceptable”. All questionnaires were completed using a pen and paper and were collected with the participant outside of VR.

### 2.6. Mastication Parameters

An output EMG (Electromyograph) was generated as the summation of activities from the masseter muscles, as measured by the surface electrodes attached to these muscles. The summed data were used for analysis. For each pizza sample, EMG signals were translated into behavioral components of mastication using the approach from previous studies [42,43]. The following outcomes were indirectly assessed by EMG signals: the number of chewing cycles (the number of peaks), chewing duration (the duration from the beginning of the first chewing cycle to the end of the last chewing cycle before the swallowing threshold), chewing rate (calculated as the ratio of the number of chewing cycles and chewing duration), and maximal bite force (indirectly measured as the maximal amplitude of the electric potential).

### 2.7. Statistical Analysis

Means and standard deviations were calculated for all study variables. A Wilcoxon signed-rank test was used to determine differences between the blank and treatment scenes. Pearson’s or Spearman’s correlation coefficients, as appropriate, were used to determine relationships between heart rate, skin temperature, and subjective presence, and masticatory parameters, food intake, and sensory perception of the pizza rolls. All statistical analysis was conducted using JMP Pro v15 (SAS, Cary, NC, USA). Statistical significance was set at *p* < 0.05.

## 3. Results

### 3.1. Participant Details

The participants had a mean age of 30 years (SD = 4, range 22–39 years) and a mean body mass index (BMI) of 23 kg m^−2^ (SD = 1, range 20–29). The study group consisted of nine males and six females. 

### 3.2. Questionnaires

Table 1 provides data regarding the participants’ responses to the questionnaires used in this present study. Baseline appetite differences between the test sessions were not significant (*p* > 0.05). The participants rated their sense of presence in the restaurant scene to be higher than the blank scene (5.0 (SD = 1.4) vs. 3.9 (SD = 1.8); (S = 45.000, *p* < 0.006)). Moreover, there were no statistically significant differences in the participants’ ratings of the palatability of the test foods (S = 16.5, *p* = 0.43), saltiness of the test foods (S = 16.5, *p* = 0.39), texture of the test foods (S = 19.0, *p* = 0.51), or the acceptability of the test foods (S = 12.0, *p* = 0.49).

### 3.3. Food Intake

The participants consumed a mean of 9.7 pizza rolls (SD = 1.03) for the blank scene and 10.3 pizza rolls (SD = 1.3) for the restaurant scene. This was equal to 340 kcal for the blank scene and 361 kcal for the restaurant scene. This difference was not statistically significant (S = 0.5, *p* = 0.98). Only one participant requested more pizza rolls and only one participant ate fewer than ten pizza rolls. 

### 3.4. Physiological Measures

Baseline differences between the treatment groups were not significant for any of the physiological measures (*p* > 0.05). Heart rate in the restaurant scene was significantly higher than in the blank scene (83 bpm (SD = 9) vs. 79 bpm (SD = 8); S = 38.0, *p* = 0.02). 

In addition, skin temperature in the restaurant scene was significantly higher than in the blank scene (33.6 °C (SD = 1.6) vs. 32.9 °C (SD = 1.3); S = −35.00, *p* = 0.048). 

### 3.5. Mastication Data

Table 2 provides data about the masticatory parameters. The participants in the restaurant treatment used fewer masticatory cycles before swallowing (S = 35.0, *p* = 0.047). There were no statistically significant differences in maximal bite force (S = −31.0, *p* = 0.08), chewing duration (S = 15.5, *p* = 0.38), or time per chewing cycle (S = 10.5, *p* = 0.7).

### 3.6. Correlations between Markers of Presence, Masticatory Parameters, and Appetite

The correlation coefficients between markers of presence, masticatory parameters, sensory evaluation of the test food, and appetite ratings are provided in Table 3 (blank scene) and Table 4 (restaurant scene). Only statistically significant results are discussed in the text.

### 3.7. Presence Correlations

There were statistically significant correlations between heart rate in the blank scene and the restaurant scene (*r* = 0.71, *p* = 0.003), skin temperature rate in the blank scene and the restaurant scene (*r* = 0.61, *p* = 0.01), and subjective presence in the blank scene and restuarant scene (*r* = 0.64, *p* = 0.008). No statistically significant correlations were found between subjective presence and heart rate or skin temperature.

### 3.8. Presence and Mastication Correlations

There were no statistically significant correlations between markers of presence in the blank scene and masticatory parameters (Table 3). In the restaurant scene (Table 4), there was a statistically significant inverse correlation between subjective presence and chewing duration (r = −0.62, *p* = 0.01).

### 3.9. Presence and Sensory Perception of the Pizza Rolls

In the blank scene (Table 3), there was a significant correlation between skin temperature and saltiness (*r*_s_ = −0.62, *p* = 0.01) and skin temperature and palatability (*r*_s_ = 0.52, *p* = 0.04). In the restaurant scene, there was a statistically significant correlation between acceptibility of the pizza rolls and skin temperature (*r*_s_ = −0.57, *p* = 0.03).

### 3.10. Presence and Appetite Correlations

In the blank scene (Table 3), there was a statistically significant correlation between subjective presence and desire to eat (*r* = 0.53, *p* = 0.03). In the restaurant scene (Table 4), there was a statistically significant correlation between subjective presence and hunger (*r* = −0.57, *p* = 0.03) and subjective presence and desire to eat (*r* = −0.55, *p* = 0.03).

## 4. Discussion

In the present study, the potential of using a VR-HMD to study food intake and eating behavior was investigated. The present study indicates that eating while wearing a VR-HMD is possible, and that food intake and eating behavior data can be collected in this way. However, we found few differences in food intake or eating behavior between the two virtual scenes, and any differences were relatively minor.

Studies of food intake or eating behavior are generally carried out in the laboratory environment. This approach has several strengths, including strong experimental control and the ability to make measurements that are invasive (e.g., to collect blood samples to measure hormones or metabolites) or require equipment that is not easily portable (e.g., equipment to measure masticatory parameters or the microstructure of eating). However, laboratory studies do not reflect the conditions under which food is typically eaten and the results may not hold in real-life situations. VR potentially provides a new medium to study meal size and eating behavior in different environments, as any eating environment can be recreated using computer graphics [44]. As with all laboratory studies, participants likely recognize that they are being observed and may make decisions that reflect the “moral choice” rather than the decision they would actually make under real-life circumstances [45,46]. This does not mean that these studies are not useful, and the results from these studies can provide insights into what could happen in a given situation. Moreover, they can also provide information about the underlying mechanisms that underpin behavior in real-life situations. Rather than being seen as a replication of or replacement for real-life studies, studies using VR should be seen as an extension of current laboratory studies that allow for behavior in different contexts to be examined, possibly providing new insights into eating behavior.

To facilitate eating while wearing a HMD, this present study used an infrared camera that modeled the movement of the user’s real hands onto the VR space so that they could locate and interact with objects in the virtual environment. Objects (table, plate, and pizza bites) in the virtual space were mapped onto the location of the same objects in the real world using Vive trackers attached to the chair and table. Thus, when the user touched the table, plate, or food in the virtual environment they touched a real-life table, plate, or pizza bite. During debriefing, participants did not report difficulty in locating the food, although placing the food in the mouth took a minor amount of practice and concentration. This may have slowed the eating process down and participants may have focused more on the amount eaten or found the eating process unusual, causing them to stop eating prematurely.

In this present study, food intake was similar in the blank and restaurant conditions. This may suggest that the eating environment has no effect on meal size, but limitations to the approach used in this present study may have masked an effect. The use of a single test food may have led to the development of sensory-specific satiety [47], so the participant ceased eating due to sensory boredom rather than physiological appetite. Sensory-specific satiety can be reduced by using different types of food, such as different types of sandwiches [48]. In the present study, when the participant picked up a pizza roll, a virtual version was removed from the plate so the participant could determine how many pizza rolls they had consumed. This approach was selected as this represented real life (i.e., people receive visual cues regarding how much they have eaten). However, the portion size of the meal presented may influence the amount eaten. For instance, the portion size presented has been shown to increase food intake [49] as individuals often clear the amount on their plate [50]. Indeed, thirteen participants consumed the full portion of pizza bites and did not request an additional serving. Alternative methods of presenting food in VR (e.g., a single piece of food at a time) and its effect on food intake or eating parameters should be investigated. Moreover, a potentially interesting use of VR is to manipulate the food’s characteristics in the virtual world (e.g., size or color) or to remove cues related to how much has been eaten to determine the effect on eating. One study found that removing visual cues regarding how much was eaten by using a “bottomless bowl” of soup markedly increased food intake [51]. VR could be used to understand how food intake is affected when sensory cues are corrupted. In addition, eating while wearing an HMD may have focused the participants’ attention on the amount eaten or they may have found the eating process unusual, causing them to stop eating prematurely. Further research is required, using different test foods and portion sizes, to determine the optimum approach that works within the confines of VR (i.e., what foods can feasibly be eaten while wearing an HMD). Alternatively, the use of CAVE systems as a method to investigate meal size may prove more useful and research into their use is warranted.

VR potentially offers a new tool to develop interesting approaches (e.g., providing conflicting sensory information or changing aspects of the eating environment (background sounds or distractions such as TVs)) to study the factors that influence eating. We found that participants made fewer masticatory cycles before swallowing in the restaurant condition compared to the blank scene. However, there were no statistically significant differences in any of the other masticatory or eating parameters. The mastication data collected appear to be plausible. A previous study using Totino’s cheese pizza rolls as a test food found that participants took approximately 30 chewing cycles before swallowing (compared to 35 in the restaurant scene or 38 in the blank scene), took approximately 27 s to chew the test food (compared to 29 s for the blank scene and 27 s for the restaurant scene), and exhibited a chewing duration of approximately 1.1 chews/second (compared to 1.3 s in both VR scenes) [43]. In the study by Zhu and Hollis [43], it was found that individuals with obesity exhibited different masticatory characteristics, and it would be interesting to study how the eating environment influences eating parameters in individuals with obesity. Due to the link between the microstructure of eating and meal size, a full investigation of how the eating environment influences eating rate, masticatory parameters, and duration of eating is warranted. VR may potentially provide a new approach to better understand how different eating environments influence the microstructure of eating. In addition, other parameters of oral processing should be investigated. In particular, the particle size of the swallowed bolus should be explored, as this may have a significant effect on postprandial appetite and metabolism [52]. As studies of the bolus characteristics require the expectoration of food before swallowing, the use of virtual environments rather than real-life restaurants would appear preferable.

VR may provide a method to study the effect of product-intrinsic and product-extrinsic factors on flavor perception. For this to be successful, individuals would need to integrate information from the virtual world with the real food being eaten and believe that they have a common source. A number of studies using VR indicate that users do integrate information from VR into their beliefs about the world [53,54]. An example that is salient to food is a recent study that found that the color of tea represented in VR was integrated with sensory cues from the real world to influence the perception of an actual drink [55]. In this study, we did not find an effect of the VR environment on the sensory perception of a food. This may have been due to participants rating the sensory characteristics shortly after eating them rather than contemporaneously. In addition, the model used to represent the test food was similar in dimensions and color but was not a photorealistic representation of the food, and this may have influenced the participants’ evaluations. In addition, we found some (although not consistent) correlations between HR and skin temperature and perception of the test foods. The link between these physiological markers and food perception warrants further attention. A recent study found that participants rated the taste of cheese differently depending on the environments experienced through an HMD [56]. The study by Stelick et al. differed from this present study as it used a 360° video of the scenes rather than a computer-generated scene. The strengths and limitations of each of these approaches to presenting VR warrants further study. In addition, the effect of introducing other sensory stimuli, such as touch and odor, into VR studies using haptics or olfactometers warrants investigation. It is likely that more sensory modalities being involved in VR studies will increase the sense of presence and the likelihood that individuals will behavior in a manner more similar to real life.

The eating environment may elicit emotions that influence meal size. In this present study, an increase in heart rate and skin temperature were observed when the participants were experiencing the restaurant scene, suggesting increased emotional arousal [57]. However, the effect on heart rate was modest; it increased from 79 BPM to 83 BPM. The practical significance of this is unclear. Several studies have found that immersing individuals in VR can elicit an emotional response [58,59,60]. Research shows that emotions can impair or facilitate decision-making [61,62], and VR may be a useful medium for investigating the role of emotions on eating behaviors or food choices in different eating environments. In the present study, correlation analysis hinted at possible relationships between feelings of presence and several masticatory parameters. Research that investigates the influence of emotion or arousal on eating parameters may yield new insights into how emotion influences food intake.

In the present study, participants rated their presence on a 7 point scale at ~4 in the blank scene and 5 in the restaurant scene. In addition, we observed an increase in heart rate and skin temperature when participants were in the restaurant scene. There were no statistically significant correlations between these measures. However, there were correlations between HR, skin temperature, and subjective presence between the scenes (i.e., people who exhibited higher presence in the blank scene also exhibited higher presence in the restaurant scene). There are several issues with using questionnaires to determine presence. For instance, participants may respond to questions in idiosyncratic ways. When participants in one study were asked the question “Please rate your sense of being in the office space,” participants in a real office space only rated it 4 on a 7 point scale [40]. Presumably, the participants recognized they were inhabiting reality, but were possibly comparing the office to their model of what an office should look like and the low score reflected the discrepancy. Consequently, it has been suggested that using questionnaires across different types of environment (e.g., immersive VR vs. desktop PC) may have limited utility [40]. Another major issue with using questionnaires is that participants may guess the purpose of the study, especially as blinding may not be possible, and provide responses to questions they believe the researchers are looking for. Consequently, there is a need to develop physiological markers of presence that provide an objective measure of presence. It is likely that the most useful physiological markers of presence in studies of restaurants mirror the physiological responses observed when an individual is in a real-life restaurant [36].

Future advances in VR technology will likely improve its suitability to study food intake and eating behavior. It is likely that headsets will become smaller and provide improved graphical representations of the world. Moreover, the development of better hand tracking would aid in locating and manipulating food. It is likely that VR will integrate haptic technologies or environmental odors into the representations of the world to further enhance presence. Other associated technologies, such as mixed reality or augmented reality, may also be used to investigate food perception or food intake. However, technological limitations such as a limited field of view may limit their usefulness at this stage.

This present study had several limitations. First, the study sample was relatively small and larger studies with adequate statistical power are required to extend these observations. In particular, studies sufficiently powered to understand gender, body mass index, and experience with VR are required. The lack of statistical power means that there is a higher risk of producing false positive results [63]. Consequently, the data should be viewed as preliminary until confirmed by larger studies. Second, the study group was homogenous and, being largely undergraduate and graduate students or university staff members, was not diverse in terms of age and background. Moreover, while we did not quantify previous exposure to VR, it is likely that the majority of the study group had used VR headsets. The previous use of VR could influence the sense of presence or physiological responses. Consequently, future studies should make efforts to understand how previous use of VR influences responses and to identify differences between experienced and naive users. Third, it is possible that participants guessed the purpose of the study, creating a demand bias wherein they rated their sense of presence higher on the questionnaire. However, we also observed increased heart rate and skin temperature, suggesting increased arousal when in the VR restaurant scene. Fourth, the results of this study only hold for pizza bites, which is a food that most people find palatable. It is possible that for other foods, for which palatability is more variable among the population, the eating environment may have larger effects on food perception and palatability. Further studies are required using different test foods. The participants also tended to eat the full portion of pizza rolls, with only one participant requesting more pizza rolls. This could have introduced a ceiling effect masking an effect on meal size. This present study used a within-subjects design and there is the risk of a carryover effect influencing the results.

Despite these limitations, this study describes a method that we successfully used to study food intake and eating behavior in VR. This approach supports other VR studies that have required individuals to eat while wearing a VR-HMD [32,64] and provides further evidence that this is a feasible approach to study eating behavior, the impact of the eating environment on the sensory perception of food [32], and potentially the effect of emotions on eating behavior and food perception [64].

## 5. Conclusions

The approach detailed in this manuscript potentially provides a new approach to understanding the influence of the eating environment of eating behavior. However, while the participants reported an increased sense of presence along with increased skin temperature and heart rate (putative physiological markers of presence), there were minimal changes in food intake, masticatory parameters, and the sensory evaluation of the pizza bites. This study provides information to aid the development of future studies using this approach to understanding how the food environment influences eating parameters or the sensory evaluation of foods.

## Figures and Tables

**Table 1:** The participants’ responses to baseline appetite questions, sense of presence, and sensory characteristics of the test food. *N* = 15 for all questions in the restaurant and blank scene.

	Blank Scene (Mean (SD))	Restaurant Scene (Mean (SD))	*p* Value
Baseline hunger (mm)	72 (22)	66 (17)	0.4
Baseline fullness (mm)	26 (20)	28 (18)	0.86
Baseline desire to eat (mm)	71 (22)	70 (16)	0.45
Baseline amount eaten (mm)	70 (17)	71 (14)	0.77
Presence ^a^ (mean) ^b^	3.9 (1.8)	5.0 (1.4)	0.006
Palatability ^c^	6.0 (1.9)	6.1 (1.2)	0.43
Saltiness ^c^	4.4 (1.3)	4.9 (1.9)	0.39
Texture ^c^	5.6 (1.5)	5.9 (1.5)	0.51
Acceptability ^c^	5.9 (1.5)	6.1 (1.4)	0.49

^a^ Presence refers to the feeling of being in the virtual environment. ^b^ The participants’ ratings of presence were captured on a 7 point scale and responses could range from 1–7. ^c^ The participants rated the sensory characteristics of the test foods on a 9 point Likert scale and responses could range from 1–9.

**Table 2:** Masticatory parameter data in the blank scene and restaurant. *N* = 15 for all measurements in both the blank and restaurant scene.

	Blank Scene (Mean (SD))	Restaurant Scene (Mean (SD))	*p* Value
Number of masticatory cycles before swallowing	38 (11)	35 (6)	0.047
Maximal bite force (mV) ^a^	0.7 (0.5)	0.9 (0.3)	0.08
Chewing duration (s)	29 (8)	27 (4)	0.38
Time per chewing cycle (s)	1.3 (0.3)	1.3 (0.2)	0.7

^a^ Maximal bite force was indirectly measured as the maximal amplitude of the electric potential.

**Table 3:** Correlation coefficients between markers of presence and masticatory parameters in the blank scene. * *p* < 0.05.

	Blank Skin Temperature	Blank Heart Rate	Blank Presence
Blank skin temperature	1	0.1	0.08
Blank heart rate	0.1	1	−0.07
Blank presence	0.08	−0.07	1
Restaurant skin temperature	0.61 *	−0.14	−0.08
Restaurant heart rate	−0.182	0.71 *	−0.11
Restaurant presence	0.003	−0.15	0.64 *
Chewing cycles	−0.16	0.25	0.28
Chewing time	−0.06	0.002	0.39
Max bite force	−0.22	0.09	−0.12
Chewing duration	0.2	0.41	−0.27
Palatability	0.52 *	−0.12	0.08
Saltiness	−0.62 *	−0.08	−0.27
Texture	0.13	0.05	0.21
Acceptability	0.39	−0.01	0.15
Hunger	−0.06	0.06	0.12
Fullness	0.23	−0.15	0.01
Desire to eat	0.08	−0.06	0.53 *
Prospective consumption	−0.08	0.02	0.08

**Table 4:** Correlation coefficients between markers of presence and masticatory parameters in the restaurant scene. * *p* < 0.05.

	Restaurant Skin Temperature	Restaurant Heart Rate	Restaurant Presence
Blank skin temperature	0.61 *	−0.18	0.03
Blank heart rate	−0.14	0.71 *	−0.15
Blank presence	−0.08	−0.12	0.64 *
Restaurant skin temperature	1	−0.18	0.08
Restaurant heart rate	−0.18	1	−0.1
Restaurant presence	−0.08	0.1	1
Chewing cycles	−0.05	0.37	−0.05
Chewing time	−0.20	0.31	0.36
Max bite force	0.15	−0.36	0.38
Chewing duration	0.16	0.07	−0.62 *
Palatability	−0.23	0.13	−0.07
Salty	−0.32	0.10	−0.18
Texture	0.22	0.24	0.11
Acceptability	−0.57 *	0.04	−0.11
Hunger	0.07	0.30	−0.57 *
Fullness	0.17	−0.34	0.35
Desire to eat	0.06	0.31	−0.55 *
Prospective consumption	0.17	0.19	−0.42

## Data Availability

The data from this study is not available as participants did not consent to data sharing.
